# Supplementary material for: CADMA-Chem: A Computational Protocol Based on Chemical Properties Aimed to Design Multifunctional Antioxidants
Source: Int J Mol Sci. 2022 Oct 31;23(21):13246. doi: 10.3390/ijms232113246 (PMC9658414; doi:10.3390/ijms232113246)
Supplement: Supplementary file 1 [file ijms-23-13246-s001.zip › ijms-1956463-supplementary.pdf]

Supporting Information

CADMA-Chem: A Computational Protocol Based on Chemical Properties Aimed to Design Multifunctional Antioxidants

Eduardo Gabriel Guzmán-López,[1] Miguel Reina,[1] Romina Castañeda-Arriaga,[1] Adriana Pérez-González,[2] Misaela Francisco-Márquez,[3] Luis Felipe Hernández-Ayala,[1] Annia Galano,[1*]

[1] *Departamento de Química. Universidad Autónoma Metropolitana-Iztapalapa. Ave. Ferrocarril San Rafael Atlixco 186, Col. Leyes de Reforma 1A Sección, Alcaldía Iztapalapa. C. P. 09310. México City. México.*

[2] *CONACYT - Universidad Autónoma Metropolitana-Iztapalapa. Ave. Ferrocarril San Rafael Atlixco 186, Col. Leyes de Reforma 1A Sección, Alcaldía Iztapalapa. C. P. 09310. México City. México.*

[3] *Instituto Politécnico Nacional- UPIICSA. Té 950, Col. Granjas México, C.P. 08400, México City. México.*

Contents

Figure S1. Complete interaction path in the [dM38–COMT] complex. 2

Table S1. Reference set of molecules, with some neuroprotective effects. 3

Table S2. Values of the ADME properties, toxicity (LD_{50} and M), synthetic accessibility (SA), the selection score (S^S), and elimination scores (S^E) for dM38 and its parent molecule (melatonin). Average values (^{A}RS) and standard deviation (^{SD}RS) for the reference set of molecules. * 5

Table S3. First ionization energy (IE), electron affinities (EA), and bond dissociation energies (BDE) for dM38, its parent molecule and reference antioxidants........ 6

Table S4. Gibbs free energies of reaction for the Cu(II) chelation by dM38 and the corresponding Maxwell-Boltzmann populations. 7

Supplementary Text S1. Selection, elimination and polygenic scores........ 8

- Acronyms: 8
- Selection score (S^S): 8
- Elimination score (S^E): 9
- Polygenic score (S^P): 10

Supplementary Text S2. Molecular docking, computational details........ 11

* E-mail: agalano@prodigy.net.mx, agal@xanum.uam.mx

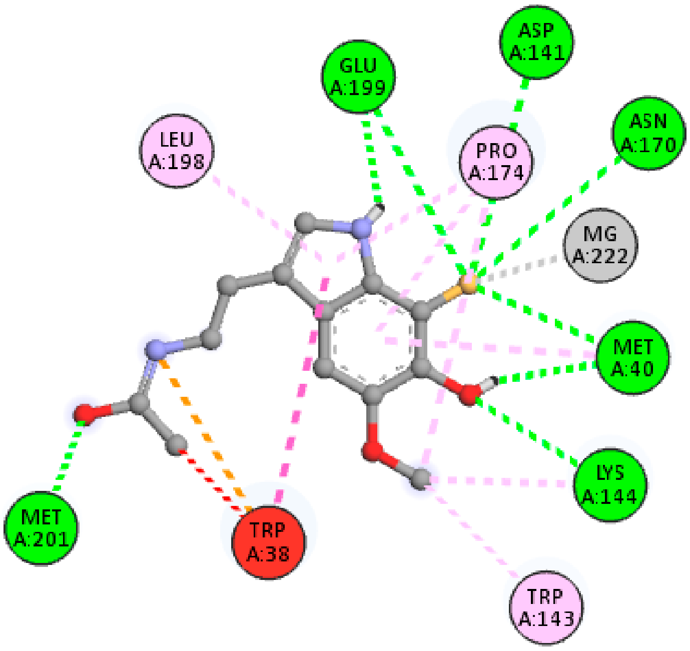

Figure S1. Complete interaction path in the [dM38–COMT] complex.

Table S1. Reference set of molecules, with some neuroprotective effects.

Compound (CAS)	Structure	Compound (CAS)	Structure
Acetylcarnitine (3040-38-8)	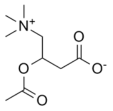	Masitinib (790299-79-5)	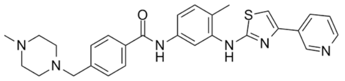
Amantadine (768-94-5)	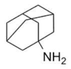	Melatonin (73-31-4)	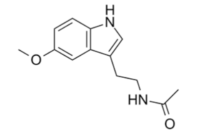
Apomorphine (58-00-4)	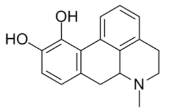	Memantine (19982-08-2)	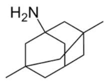
Baclofen (1134-47-0)	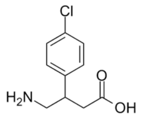	Modafinil (68693-11-8)	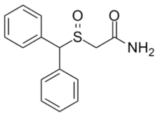
Benserazide (14919-77-8)	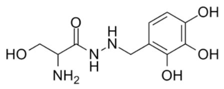	Piribedil (3605-01-4)	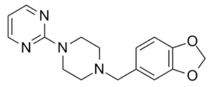
Benztropine (86-13-5)	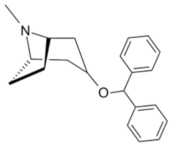	Pramipexole (104632-26-0)	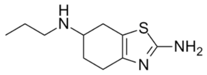
Biperiden (514-65-8)	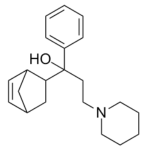	Procyclidine (77-37-2)	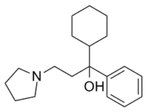
Bromocriptine (25614-03-3)	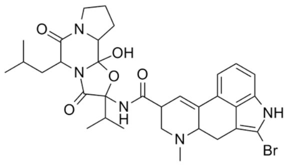	Remacemide (128298-28-2)	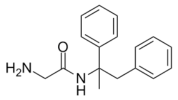
Cabergoline (81409-90-7)	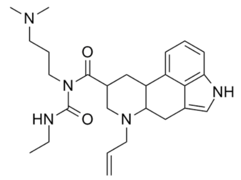	Riluzole (1744-22-5)	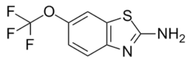
Carbidopa (28860-95-9)	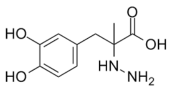	Rivastigmine (123441-03-2)	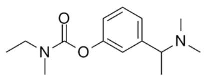

Curcumin (458-37-7)	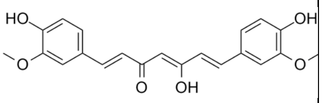	Ropinirole (91374-21-9)	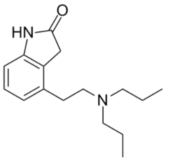
Dantrolene (7261-97-4)	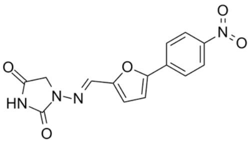	Selegiline (14611-51-9)	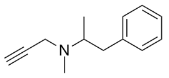
Donepezil (120014-06-4)	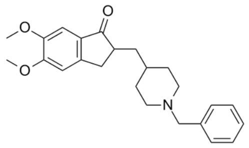	Tacrine (321-64-2)	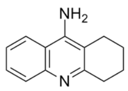
Entacapone (130929-57-6)	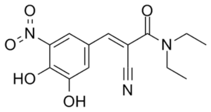	Tetrabenazine (58-46-8)	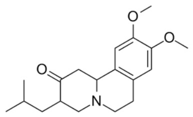
Galantamine (357-70-0)	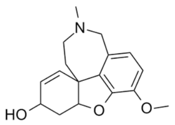	Tizanidine (51322-75-9)	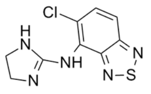
Ladostigil (209349-27-4)	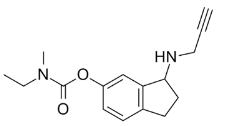	Tolcapone (134308-13-7)	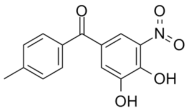
L-DOPA (59-92-7)	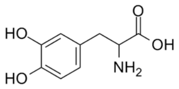	Trihexyphenid yl (144-11-6)	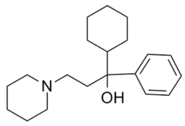
Lisuride (18016-80-3)	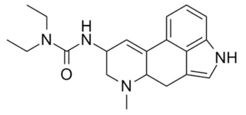		

Table S2. Values of the ADME properties, toxicity (LD_{50} and M), synthetic accessibility (SA), the selection score (S^S), and elimination scores (S^E) for dM38 and its parent molecule (melatonin). Average values (^{A}RS) and standard deviation (^{SD}RS) for the reference set of molecules. *

	**dM38****		**Melatonin****		**^{A}RS**	**^{SD}RS**
	**Value**	S_i^E	**Value**	S_i^E		
logP	1.12	0.437	1.45	0.289	2.090	2.230
MW	280.35	0.065	232.28	0.563	286.637	96.571
PSA	74.35	0.404	54.12	0.156	59.745	36.135
^{X}A	19	0.229	17	0.526	20.543	6.736
HB^A	5	0.297	4	0.136	4.314	2.311
HB^D	3	0.631	2	0.050	1.914	1.721
RB	4	0.014	4	0.014	3.971	2.079
^{M}R	79	0.117	69.38	0.465	82.255	27.709
LD_{50}	664.47	0.354	1298.11	0.403	960.771	836.369
M	0.02	0.304	0.05	1.204	0.412	0.299
SA	3.81	0.322	2.46	1.550	4.161	1.094
S^S	3.61		3.75		3	
$S^{E,ADME2}$		0.502		0.852		
$S^{E,ADME8}$		2.194		2.199		
$S^{E,ADMET}$		2.852		3.806		
$S^{E,ADMETSA}$		3.174		5.356		

* logP= Octanol/water partition coefficient. PSA= Polar surface area. ^{X}A= Number of non-hydrogen atoms. MW= Molecular weight. HB^A= Number of acceptors in H-bond interactions. HB^D= Number of donors in H-bond interactions. ^{M}R= Molar refractivity. LD_{50}= Oral rat 50 percent lethal dose. M= Ames mutagenicity. SA= Synthetic accessibility. $S^{E,ADME2}$, $S^{E,ADME8}$, $S^{E,ADMET}$, and $S^{E,ADMETSA}$ defined in Appendix 2S.

** $S_i^E = \left|\frac{P_{i,\overline{RefSet}} - P_{i,dM38}}{SD(P_i)}\right|$ where Pi represents each property, and SD is the corresponding standard deviation for the reference set of molecules.

Table S3. First ionization energy (IE), electron affinities (EA), and bond dissociation energies (BDE) for dM38, its parent molecule and reference antioxidants.

	Species	**IE** (eV)	**EA** (eV)	**BDE** (kcal/mol)
Melatonin	neutral	7.49	-0.97	89.33
dM38	neutral	7.17	-0.93	71.51
	Mono-anion	1.95	-2.70	68.31
Trolox	Mono-anion	4.32	-3.07	76.05
Ascorbic acid	Neutral	9.13	-0.63	78.87
	Mono-anion	3.29	-4.16	69.64
α-tocopherol	Neutral	7.26	-1.16	84.15

Table S4. Gibbs free energies of reaction for the Cu(II) chelation by dM38 and the corresponding Maxwell-Boltzmann populations.

	**ΔG, pH=7.4** (kcal/mol)	**MB** (%)
DCM-cS	-20.00	4.08E-09
DCM-cSO	-28.45	0.0064
DCM-cSN	-16.16	6.26E-12
CDCM-cSO	-34.17	99.9927
CDCM-cSN	-27.26	0.0009

Supplementary Text S1. Selection, elimination and polygenic scores

Acronyms:

^{X}A: Number of non-hydrogen atoms.

HBA: Number of acceptors in H-bond interactions.

HBD: Number of donors in H-bond interactions.

LD_{50}: Oral rat 50 percent lethal dose.

logP: Octanol/water partition coefficient.

M: Ames mutagenicity.

MR: Molar refractivity.

MW: Molecular weight.

PSA: Polar surface area.

RB: Number of rotatable bonds.

SA: Synthetic accessibility.

Selection score (S^S):

The selection score was designed to identify the candidates with higher drug-like behavior. It includes the physicochemical parameters necessary to evaluate ADME properties (S^{ADME}), as well as toxicity (S^{T}) and synthetic accessibility (S^{SA}):

$$S^S = S^{ADME} + S^T + S^{SA}$$

where

$$S^{ADME} = \frac{S^{logP} + S^{HBD} + S^{HBA} + S^{MW} + S^{MR} + S^{XA} + S^{RB} + S^{PSA}}{8}$$

$$S^T = \frac{S^{LD_{50}} + S^M}{2}$$

The following terms will be 1 or 0 depending on the particular requirements:

$$S^{logP} = \begin{cases} 1, if \ -0.4 \le logP \le 5.0 \\ 0, otherwise \end{cases}$$

$$S^{HBD} = \begin{cases} 1, if\ HBD \le 5 \\ 0, otherwise \end{cases}$$

$$S^{HBA} = \begin{cases} 1, if\ HBA \leq 10 \\ 0, otherwise \end{cases}$$

$$S^{MW} = \begin{cases} 1, if\ 160 \leq MW \leq 480 \\ 0, otherwise \end{cases}$$

$$S^{MR} = \begin{cases} 1, if\ 40 \leq MR \leq 130 \\ 0, otherwise \end{cases}$$

$$S^{XA} = \begin{cases} 1, if\ AtX \leq 70 \\ 0, otherwise \end{cases}$$

$$S^{RB} = \begin{cases} 1, if\ RB \leq 10 \\ 0, otherwise \end{cases}$$

$$S^{PSA} = \begin{cases} 1, if\ PSA \leq 140 \\ 0, otherwise \end{cases}$$

$$S^{LD_{50}} = 1 + log\left(\frac{{LD_{50}}^{dCHA}}{{LD_{50}}^{\overline{RefSet}}}\right)$$

$$S^{M} = 1 + log\left(\frac{M^{\overline{RefSet}}}{M^{dCHA}}\right)$$

$$S^{SA} = 1 + log\left(\frac{SA^{\overline{RefSet}}}{SA^{dCHA}}\right)$$

Elimination score (S^E):

To verify if any chalcone derivative deviates significantly from the average value of the reference set, in any of the properties analyzed, four elimination scores (S^E)[87, 98, 99] were used:

1° $S^{E,ADME2}$, includes log P y MW:

$$S^{E,ADME2} = \left|\frac{logP_{\overline{RefSet}} - logP_{dCHA}}{SD_{logP}}\right| + \left|\frac{MW_{\overline{RefSet}} - MW_{dCHA}}{SD_{MW}}\right|$$

2° $S^{E,ADME8}$, includes 8 terms, one for each ADME property:

$$S^{E,ADME8} = S^{E,ADME2} + \left|\frac{PSA_{\overline{RefSet}} - PSA_{dCHA}}{SD_{PSA}}\right| + \left|\frac{{}^{X}A_{\overline{RefSet}} - {}^{X}A_{dCHA}}{SD_{AtX}}\right| + \left|\frac{HBA_{\overline{RefSet}} - HBA_{dCHA}}{SD_{HBA}}\right| + \left|\frac{HBD_{\overline{RefSet}} - HBD_{dCHA}}{SD_{HBD}}\right| + \left|\frac{RB_{\overline{RefSet}} - RB_{dCHA}}{SD_{RB}}\right| + \left|\frac{MR_{\overline{RefSet}} - MR_{dCHA}}{SD_{MR}}\right|$$

3° $S^{E,ADMET}$, includes the toxicity:

$$S^{E,ADMET} = S^{E,ADME8} + \left|\frac{LD_{50\overline{RefSet}} - LD_{50dCHA}}{SD_{LD_{50}}}\right| + \left|\frac{M_{\overline{RefSet}} - M_{dCHA}}{SD_{M}}\right|$$

4° $S^{E,ADMETSA}$, includes the term of synthetic accessibility.

$$S^{E,ADMETA} = S^{E,ADMET} + \left|\frac{SA_{\overline{RefSet}} - SA_{dCHA}}{SD_{SA}}\right|$$

Polygenic score (S^P):

The binding energies obtain from the docking studies are used to construct this index, which was designed to evaluate the multi-target ligand behavior of the candidates (for example, melatonin derivatives: dmX). The score takes the natural substrate of each enzyme as reference:

$$S^{P} = \frac{\Delta G^{COMT}_{U,\,dmX}}{\Delta G^{COMT}_{U,dopamine}} + \frac{\Delta G^{MAOB}_{U,\,dmX}}{\Delta G^{MAOB}_{U,phenylethylamine}} + \frac{\Delta G^{AChE}_{U,\,dmX}}{\Delta G^{AChE}_{U,\,acetylcholine}}$$

Supplementary Text S2. Molecular docking, computational details.

The crystal structures of catechol-o-methyl transferase (COMT, PDB ID: 4PYL[121]), monoamine oxidase B (MAO-B, PDB ID: 2V5Z)[122] and acetylcholinesterase (AChE, PDB ID: 4EY7)[123] co-crystallized with tolcapone, safinamide and donepezil, respectively, were obtained from the protein data bank. All structures were modelled considering *p*H=7.4. Prior to docking experiments, enzymes were prepared as follows. Monomers, water molecules, non-interest species (DMSO, Cl-, ethyleneglycol, etc.) and inhibitors were removed. Then, polar hydrogens and Kollman charges were added using Chimera 1.16 software.[124] The optimized structures of all ligands were saved to .mol2 files; atomic charges estimated by DFT protocol (M05-2X/6-311+G(d,p)) were added manually. The docking studies were carried out using AutoDock Vina software.[125] A Lamarckian genetic algorithm study was performed inside the protein-ligand complexes centered at x: -10.49, y: 40.60, z: 61.64 and grid size of 20 x 20 x 20 Å3 for COMT, x: 53.81, y: 156.34, z: 28.14 and grid size of 13 x 13 x 13 Å3 for MAO-B and x: -14.00, y: -43.64, z: 27.10 and grid size of 15 x 13 x 13 Å3 for AChE. 150 individual steps in population with 2.5 x 10^4 evaluations result in 10 docked poses. For the most stable conformation, docking scores (free binding energy, ΔG_U) is reported. Inhibition constant values were calculated as Ki=e-(ΔG/RT). Finally, the best docked conformation was analyzed with Discovery Studio 2021 graphic interface.[126] The RMSD values for the best possess of tolcapone (COMT), safinamide (MAOB) and donepezil (AChE) were 1.2, 0.9 and 1.4 Å respectively. The estimated docking scores correlated well with experimental observations. These findings confirm that the docking methodology is suitable to reproduce the binding mode of the studied compounds with the target biomolecules.
